# Harnessing the early-life gut microbiome for sustainable ruminant production

**DOI:** 10.1186/s42523-026-00549-6

**Published:** 2026-03-17

**Authors:** Limei Lin, Milka Popova, Ilma Tapio, Le Luo Guan, Jana Seifert

**Affiliations:** 1 Faculty of Land and Food Systems, The University of British Columbia, Vancouver, BC V6T 1Z4 Canada; 2 Université Clermont Auvergne, INRAE, VetAgro Sup, UMR Herbivores, Saint-Genès-Champanelle, France; 3 Production Systems, Genomics and Breeding, Natural Resources Institute Finland (Luke), Jokioinen, 31600 Finland; 4 Department of Functional Microbiology of Livestock, University of Hohenheim, 70593 Stuttgart, Germany; 5 HoLMiR—Hohenheim Center for Livestock Microbiome Research, University of Hohenheim, 70599 Stuttgart, Germany

**Keywords:** Young ruminants, Early life microbiome, Animal nutrition, Husbandry

## Abstract

The gut microbiome is a dynamic and complex ecosystem that plays an important role in determining ruminant health, productivity, and environmental footprint. Its plasticity during early life presents a critical window for potential programming of long-term physiological and functional capacities, with early interventions demonstrating greater effectiveness compared to those applied in the stable adult microbiome. This review synthesizes current evidence on the assembly of the early-life gut microbiome, elucidates the mechanisms and effectiveness of various interventions (including nutritional, microbial, and genetic approaches), and evaluates their impacts on feed efficiency, animal health, and methane emissions. While various intervention strategies offer promise for advancing ruminant production, challenges remain due to the inconsistency and variability in the persistence and long-term efficacy of these protocols. We highlight the urgent need for integrated and standardized intervention strategies, alongside longitudinal, multi-omics research, and the application of artificial intelligence, to fully unlock the transformative potential of early-life microbiome modulation. Such efforts are essential to aligning ruminant production systems with global food security and climate sustainability goals.

## Introduction

Ruminants play a vital role in global food systems by supplying high-quality protein in the form of meat, milk, and co-products. However, the livestock industry faces mounting challenges related to enhancing feed efficiency, improving reproductive performance, maintaining animal health, and mitigating environmental impact [1, 2]. These challenges arise from complex interactions among host genetics, physiology, management practices, and the microbiome. The gut microbiome is a complex and dynamic microbial ecosystem that operates within a host-regulated ecosystem to mediate nutrient transformation, interact with host immune processes, and influence greenhouse gas emissions. Consequently, the gut microbiome is a pivotal determinant of both animal productivity and environmental sustainability [2]. The gut microbial ecosystem is highly resilient, and once established, the adult microbiome remains remarkably stable, limiting the effectiveness of most interventions to transient effects [3–6]. Perturbations arising from dietary modification or exogenous microbial introduction generally induce short-lived shifts, with the resident community structure typically re-establishing within weeks [3]. This ecological resilience is largely attributed to functional redundancy, a defining characteristic of microbial ecosystems wherein multiple, taxonomically distinct organisms can perform overlapping enzymatic and metabolic functions, acting on similar substrates and generating comparable metabolic outputs, thereby preserving overall stability and functional capacity despite fluctuations in community composition [7, 8].

In contrast, interventions applied during early life, when the gut microbiome is still undergoing ecological assembly (i.e., community establishment and succession) and the host remains in the pre-ruminant phase, can yield more durable effects. Although most early-life interventions have traditionally focused on rumen development, emerging evidence indicates that microbial communities along the entire gastrointestinal tract (GIT), including the hindgut, are concurrently shaped during this period and may play underappreciated roles in immune maturation and physiological development [9]. In young calves [10, 11], lambs [12], and goat kids [13, 14], inoculation with exogenous rumen or fecal microbiota can accelerate microbial ecosystem maturation. These interventions have been associated with improved growth performance, earlier development of fibrolytic capacity, and enhanced immune function, which are hallmarks of a functionally mature gut ecosystem. However, evidence for durable or permanent programming effects remains inconsistent. A number of studies report that early-life microbial interventions fail to produce sustained compositional, functional or metabolic difference [15–18], with microbial communities converging toward host- and diet-specific configurations during later development. Even when early interventions induce detectable shifts during the pre-weaning period, these effects often diminish as dietary transitions, host physiological maturation, and ecological filtering reshape the gut microbiome. Although taxonomic differences between treated and untreated animals during early growth are often subtle or inconsistent [19], post-weaning analyses of microbial interaction networks indicate that early exposure to complex microbial inocula can leave a detectable ecological imprint [20]. This suggests that the long-term consequences of such interventions may be mediated less by persistent compositional shifts and more by the reorganization of microbial interactions and the establishment of stable functional consortia. The concept of microbiome programming [4] highlights the importance of targeting this early-life window, ideally within the first month after birth, before the microbial community structure reaches stabilization. During this formative period, foundational microbial interactions are still being assembled. Colonization by key functional groups, including fibrolytic and methanogenic populations, can occur within days postpartum, preceding the introduction of solid feed [21–23]. The extent to which these early interactions translate into long-term functional outcomes likely depends on multiple factors, including inoculum complexity, host genetics, diet, management practices, and the strength of subsequent ecological convergence.

A variety of early-life strategies, including nutritional, microbial, genetic, and management-based approaches, have been explored to guide the sequential and functional assembly of the gut microbiome. These strategies aim to enhance animal health, improve feed efficiency and productivity, and reduce methane emissions. Initial colonization generally begins with facultative anaerobes, which create conditions favorable for the subsequent establishment of strict anaerobes such as bacteria, archaea, fungi, and protozoa [4, 24, 25]. Both vertical (maternal) and horizontal (environmental) transmission pathways shape this process. While early microbial exposure can promote functional maturation and potentially influence long-term traits such as feed efficiency and health, outcomes are often variable and context-dependent. Furthermore, natural inoculation routes offer less control over microbial composition, requiring a balance between ecological realism and targeted intervention.

This review synthesizes current knowledge on early-life modulation of the gut microbiome in ruminants, first exploring microbial community assembly, then evaluating the mechanisms underlying diverse intervention strategies and their consequences for productivity, health, and environmental sustainability, and finally discussing future challenges and perspectives for microbiome-based precision interventions. A comprehensive understanding of the early-life gut microbiome, including its developmental dynamics and the progress made in manipulating its assembly, provides a critical foundation for designing targeted modulation strategies that can inform precision interventions aimed at enhancing ruminant performance, promoting animal health, and mitigating environmental impacts.

## Early life gut microbiome assembly

The establishment of the gut microbiome is a dynamic and sequential process that forms the foundation for lifelong digestive, immune, and metabolic functions in ruminants [25]. The assembly of the rumen microbiome is initiated by the rapid colonization of facultative anaerobes, primarily *Streptococcus* (Bacillota) and *Enterococcus* (Bacillota), which dominate within hours of birth, rapidly scavenging oxygen and thereby creating an anaerobic environment [21, 22]. These abundant pioneer species alter the physicochemical conditions of the rumen, creating an environment that supports the proliferation and establishment of obligate anaerobes such as *Segatella* and *Xylanibacter* (both formerly classified within *Prevotella*, Bacteroidota), *Ruminococcus* (Bacillota), and *Fibrobacter* (Fibrobacterota) bacteria, which become prominent by 2–4 days and reach near-adult abundance by two weeks [21, 26–28]. In parallel, methanogenic archaea, the sole biological producers of methane, appear as early as 2–4 days in lambs and calves and achieve adult-like concentrations by 10–14 days [29–31]. Notably, both fibrolytic bacteria and methanogens colonize the rumen well before the ingestion of solid feed [31], suggesting their early establishment occurs in the absence of their primary substrates. This raises important questions about their initial ecological roles, which may involve cross-feeding and hydrogen-dependent interactions driven by early fermentation processes, even at minimal substrate levels. Beyond bacteria and archaea, eukaryotic microorganisms also establish early in life. Ciliate protozoa appear within two weeks, with smaller *Entodinium* species appearing before larger endomorphs and holotrichs [32, 33]. Anaerobic fungi like *Neocallimastix*, though low in abundance but functionally important, colonizes by 8–10 days in flock-reared lambs, marking the establishment of complete fermentative capacity [21]. These pioneer taxa establish metabolic interactions, forming foundational cross-feeding networks that foster a biochemical niche conducive to subsequent microbial recruitment [34]. In parallel, gut viral communities also undergo strong life-stage–dependent shifts, transitioning from low-diversity, temperate-dominated assemblages in neonates to more diverse, lytic-dominated communities in adults, suggesting that phage-driven top–down control may contribute to microbial succession and community restructuring during early life [35]. Although the ontogeny of the rumen virome itself has not yet been systematically characterized, the tight coupling between bacteriophages and their bacterial hosts suggests that similar phage-mediated dynamics are likely to operate during early rumen microbial succession, highlighting an important priority for future research in neonatal ruminants. With the introduction of solid feed in the first or second week of life, the availability of new substrates initiates a shift in rumen microbial community assembly, favoring fibrolytic and fermentative taxa [31, 36]. Evidence from lamb and calf studies further indicates that the timing of starter feeding can modulate developmental outcomes, with initiation at 14 days of age optimizing growth performance, gut function, immune responses, and the establishment of beneficial microbial populations compared with later introduction [37, 38]. In addition to timing, the type of solid feed also shapes rumen microbial structure, as corn–soybean-based diets are associated with enrichment of bacterial communities, whereas alfalfa hay feeding favors a greater relative abundance of rumen eukaryotes [39]. Collectively, these dietary transitions mark a shift from an initial colonization phase to a functionally specialized ecosystem [40]. As solid feed intake becomes predominant, the rumen microbiome becomes functionally effective in fiber degradation and volatile fatty acid (VFA) production, although full ecological stabilization and structural maturation continue to progress over several months post-weaning [4, 16, 25]. During this period, microbial syntrophic interactions support efficient hydrogen flux management and reinforce overall fermentation efficiency [41, 42]. Overall, the trajectory from early colonization to functional maturity is a progressive, interaction-driven process that is sensitive to both developmental timing and environmental inputs.

### Factors shaping early life gut microbiome assembly

Microbiome assembly is the ecological process by which microbial communities are established, structured, and functionally organized in a given ecosystem. This process is critical because it determines whether the system consolidates into a stable, efficient ecosystem or remains susceptible to dysbiosis. Assembly encompasses which taxa colonize and when, how they interact, and how their metabolic networks support long-term functionality, yet the mechanisms governing gut assembly remain comparatively underexplored. Current evidence indicates that both vertical (maternal) and horizontal (environmental) inputs shape the earliest stages of colonization, yet how these inputs integrate to drive ecological stabilization and functional maturation is still poorly defined.

Building on this framework, early gut colonization is influenced by multiple transmission routes and management factors [43]. Vertical transmission via maternal fluids, including exposure to the birth canal, colostrum, milk, and skin, plays a pivotal role in seeding the neonatal microbial community [44–46]. These maternal sources help establish a nascent core microbiome prior to weaning [47]. In parallel, horizontal and environmental transmission, through direct contact with adult animals as well as indirect exposure via bedding, feed, and water, rapidly increases microbial diversity in the neonate [48]. Interventions using probiotics, prebiotics, or antibiotics can modulate colonization patterns to promote growth and immune development [49, 50]. However, such interventions may also result in unintended disruptions, including microbial imbalances and reduced resilience [51–53]. Disease perturbations such as neonatal diarrhea can disrupt early assembly and precipitate dysbiosis [54, 55]. Clarifying the relative contributions and interactions of these drivers over time is essential for designing early-life strategies that reproducibly support healthy maturation and durable improvements in animal performance.

## Mechanisms and strategies of early life interventions

### Vertical transmission and factors influencing microbial legacy

*Birth-associated microbial transmission* In ruminants, vertical transmission of microbes is closely linked to the natural mother–offspring bond, in which grooming, nursing, and close physical contact facilitate the early transfer of maternal microorganisms to the newborn. Although vertical transmission is primarily associated with birth and postnatal maternal contact, some studies suggest that microbial colonization of the GIT may begin in utero through exposure to microbes in amniotic fluid or placental tissues [44, 56]. Although sequence-based studies have detected microbial taxa with metabolic potential in fetal samples, demonstrating their functional establishment would require more rigorous contamination-controlled sampling and independent multi-omics validation. Overall, current evidence indicates that parturition constitute the primary drivers of initial microbiome establishment in ruminants.

During parturition, passage through the birth canal exposes the neonate to maternal vaginal and fecal microbes, initiating rumen colonization. The vaginal microbiome may act as a reservoir for key fibrolytic bacteria and methanogenic archaea, potentially contributing metabolically important taxa to the neonatal GIT. Several taxa typical of the adult rumen, including the Rikenellaceae RC9 group (Bacteroidota), *Ruminococcus*, *Butyrivibrio* (Bacillota), *Methanobrevibacter ruminantium* (Methanobacteriota), and members of Lachnospiraceae (Bacillota) and Prevotellaceae (Bacteroidota), have been detected in the vaginal microbiota [45, 46, 57]. Mode of delivery influences microbial acquisition in other species, and cesarean section can disrupt initial gut colonization [58]. Although dystocia and surgical delivery are relatively rare in ruminants, typically affecting less than 5% of births except in certain intensive breeding systems [59], their effects on vertical microbial transmission remain largely unexplored. In one study comparing cesarean-delivered and vaginally born calves, 1,163 unique microbes were identified in cesarean-delivered calves and 2,239 in vaginally delivered calves, with several taxa remaining associated with delivery mode later in life, indicating a persistent influence of early exposure [60]. These findings suggest that alterations in the maternal vaginal microbiome may exert long-term effects on the development of the offspring microbiome.

*Maternal contact and environmental exposure* Beyond parturition, sustained maternal contact and environmental exposure sustained maternal contact and environmental exposure represent additional sources of early microbial inheritance. In cow–calf and pasture-based systems, maternal feces constitute an important potential source of inoculum, as calves maintain frequent contact with the dam and lying areas. Under such conditions, maternal feces have been reported to contribute to the early establishment of the calf microbiota, with microbial signals detectable in some cohorts up to weaning [61]. However, the magnitude and persistence of this contribution appear to be strongly influenced by management practices, and are likely reduced in intensive dairy systems where calves are separated from the dam immediately after birth. Based on 16 S rRNA amplicon sequencing at the ASV level, the fecal microbiota of four-week-old calves was found to be compositionally more similar to the oral microbiota of both calves and adult cows than to the fecal microbiota of adult cows [62]. This pattern suggests frequent oral–gastrointestinal microbial exchange facilitated by behaviors such as early rumination, grooming, and social contact. Complementary quantitative metagenomic analyses further indicate that the maternal rumen microbiota constituted an important source of calf-colonizing taxa, contributing approximately 7.9% of bacterial and 49.7% of archaeal communities, relative to other potential sources such as the oral cavity, milk, and teat skin [63]. Consistent with these observations in calves, recent evidence from lambs further demonstrates that the maternal rumen microbiota plays a direct role in shaping the offspring rumen microbiota [64].

*Colostrum- and milk-mediated influences* Postnatal microbial inoculation is further facilitated by the ingestion of colostrum and milk, which supply nutrients, immune components, and viable microbes. Fresh colostrum harbors a distinct microbial community predominantly composed of Pseudomonadota (Proteobacteria), Bacillota (Firmicutes), and Bacteroidota (Bacteroidetes) [45, 65, 66]. However, its composition differs markedly from the rumen and lower GIT microbiota of calves during the first days of life [46], and only a limited number of shared operational taxonomic units (OTUs) have been observed between colostrum and fecal communities [45]. Evidence further indicates that, although a small number of microbial taxa can be directly transferred from colostrum to the GIT, these colostrum-derived populations are transient and do not persist beyond the pre-weaning period [65]. Together, these observations suggest that a specific but short-term microbial inoculum contributes to colonization during early life, rather than serving as a major source of long-term gastrointestinal colonizers via vertical transmission.

While colostrum may not serve as a principal source of microbial inoculum, its immunomodulatory role in early gut development is paramount. Timely colostrum feeding within 12 h increases *Bifidobacterium* (Actinomycetota) abundance in the developing microbiota of calves, whereas delays reduce microbial diversity and increase the risk of dysbiosis [67, 68]. Reduced *Bifidobacterium* levels are associated with immune dysregulation in dysbiotic calves [69–71]. *Bifidobacterium* is abundant from birth until weaning and is considered a keystone taxon due to its influence on community composition and activity, although its prevalence declines with age [72, 73]. Mechanistically, *Bifidobacterium* promotes immune regulation by inducing regulatory T cells, influencing microRNAs involved in tissue development, and modulating cytokines to reduce inflammation [74–76]. Its metabolism of milk oligosaccharides via ATP-binding cassette transporters and glycoside hydrolases, including fucosidases and sialidases, produces acetate and lactate that lower gut pH and inhibit pathogens [74, 77–80]. In addition, *Bifidobacterium* upregulates mucin and tight junction proteins and competes for adhesion sites, thereby strengthening the gut barrier [81, 82]. Together, these mechanisms suggest a potential stabilizing effect on the neonatal gut microbiome and may mitigate dysbiosis triggered by maternal separation, antibiotics, or dietary change.

Collectively, vertical transmission routes seed the neonatal microbiome and shape the trajectory of gut microbial development. The success and stability of this early colonization are further influenced by maternal condition. Maternal health is central both before and after birth. Metabolic disorders such as ketosis can disrupt the early microbial legacy, with associations to reduced fetal growth, underdeveloped rumen papillae, and shifts in maternal and neonatal gut microbiomes [83, 84]. Nevertheless, peripartum nutritional and microbial interventions can improve maternal status and, in turn, enhance neonatal development and colonization. Peripartum diets enriched with fermentable carbohydrates, balanced protein, and specific probiotics have been reported to improve maternal energy balance, increase the abundance and diversity of beneficial microbes in the maternal rumen, and enhance colostrum quality [85, 86]. These improvements are associated with colonization by health-associated taxa in offspring, and controlled studies show that calves born to dams receiving such interventions exhibit greater weight gain, stronger immune markers, and more stable early-life microbial succession [87]. Therefore, improving maternal nutrition before and after parturition, as well as the microbiome, is a practical strategy for enhancing the early microbial resilience, immunity and growth of young ruminants.

## Horizontal transmission and factors influencing microbial legacy

Beyond vertical transmission, horizontal transmission from the environment and conspecifics plays an equally crucial role in the ongoing diversification and functional maturation of the microbiome. Microbial horizontal transmission occurs through contact with other animals, the environment, and the ingestion of feed and water. These sources diversify the neonatal microbiome and support continued development and stabilization beyond maternal influence. Lambs reared with their mothers in flock settings acquire cellulolytic bacteria more rapidly than lambs kept with their mothers in isolation, underscoring the importance of broader environmental and social exposure [21]. Transmission of rumen protozoa may occur through shared water sources, but also by direct animal-to-animal contact, contaminated feed, or feces [88]. Where vertical transmission is constrained, as in artificially reared systems, horizontal transmission likely plays an even more critical role in early colonization and in shaping microbiome trajectories. Calves, lambs, and goat kids reared without adult contact often exhibit lower diversity and delayed maturation of the rumen microbiota compared with those reared with their mothers or alongside adult companions [10, 89, 90]. These differences generally diminish after weaning as environment and diet increasingly drive assembly [10, 90]. This highlights that while maternal contact provides essential vertical transmission for the initial seeding of the rumen microbiome, horizontal transmission from the environment and conspecifics emerges as a complementary and equally critical driver of community diversification and functional maturation over time.

## Microbial and dietary interventions

Beyond the maternal legacy, targeted microbial and nutritional interventions during early life can guide rumen microbiome assembly. Inoculation of newborn calves with mature rumen fluid from healthy donors accelerates colonization by fibrolytic, proteolytic, and methanogenic taxa and fosters a more adult-like bacterial community that improves fermentation and nutrient utilization [13, 91, 92]. Probiotics such as *Lactobacillus* and *Bifidobacterium*, administered through maternal diets or directly to calves, can reduce pathogen colonization, stimulate immune development, and strengthen gut barrier function [93]. Prebiotics including inulin and galacto-oligosaccharides selectively support beneficial endogenous microbes, enhancing fermentation and microbial protein synthesis. In calves supplemented with galacto-oligosaccharides from birth to 28 days and then either stopped or continued to 70 days, persistent shifts in rumen microbiota were observed compared with unsupplemented controls [94]. Supplementation with live yeast (*Saccharomyces cerevisiae CNCM I-1077*) and yeast metabolites in artificially reared lambs also influenced community establishment, enhancing key fiber-degrading taxa such as *Fibrobacter succinogenes*, favoring colonization of eukaryotic families including Trichostomatia and Neocallimastigaceae, and increasing genes encoding hemicellulases and cellulases, consistent with improved fiber-degrading capacity [95]. These studies indicate that early-life prebiotic and probiotic interventions can induce lasting shifts in gut communities and support improved fermentation beyond the supplementation period. However, the persistence of introduced microbes, particularly after weaning, is often constrained by microbial community convergence, whereby distinct early-life microbial assemblages become increasingly similar in taxonomic and functional composition under conventional diets and shared environments [13, 16]. Their effectiveness is determined by multiple factors, including the timing of intervention, donor–recipient microbiome compatibility, baseline diet, host genetics, environmental conditions, and the natural developmental trajectory of young animals, which encompasses age-related shifts in dietary requirements and composition.

### From early microbial modulation to host productivity

**Fig. 1:**
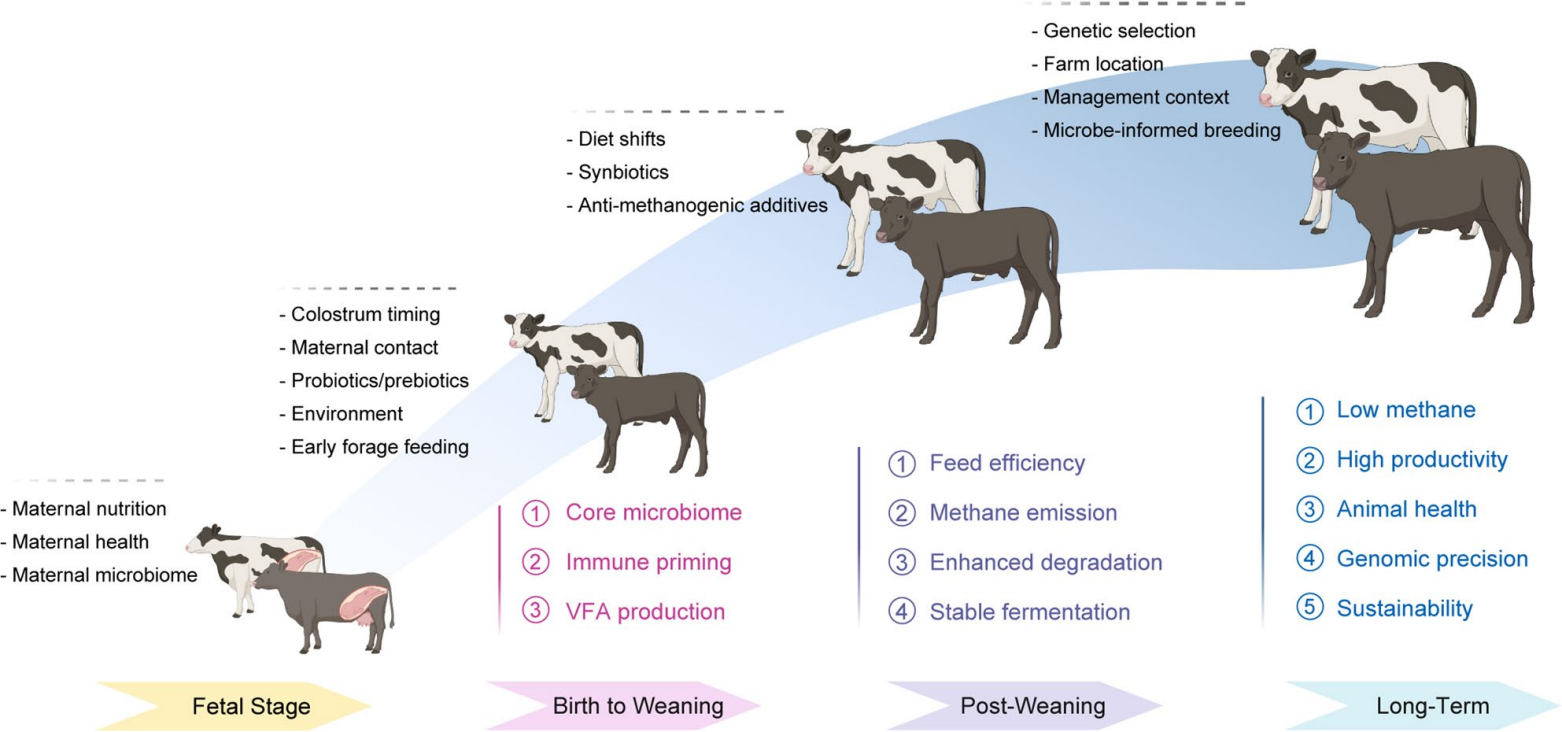
Developmental and management factors shaping the ruminant gut microbiome and downstream functional outcomes. The diagram summarizes key influences on microbial community establishment and function from the fetal stage through birth, weaning, post-weaning, and into long-term productivity. Early-life factors, including maternal nutrition, health, and microbiome composition, affect initial microbial exposure and immune priming in the fetus (1). After birth, colostrum timing, maternal contact, probiotic or prebiotic supplementation, environmental exposure, and early forage feeding modulate microbial succession and volatile fatty acid (VFA) production (2). During the post-weaning phase, dietary transitions, synbiotics (combinations of probiotics and prebiotics), and anti-methanogenic additives further influence feed efficiency, fermentation stability, and methane emission (3). Long-term outcomes are shaped by genetic selection, farm location, management practices, and microbe-informed breeding strategies, which together enhance animal health, productivity, genomic precision, and sustainability (4). Created in BioRender

A growing body of research suggests that early-life nutritional and microbial interventions can influence adult performance traits in ruminants, including growth, feed efficiency, and milk yield (Fig. 1). However, the extent to which these long-term effects are directly mediated by early-life rumen microbiome reprogramming remains unclear. Current understanding of how early microbial shifts contribute to lifelong performance outcomes is limited, and the mechanisms linking early microbiome modulation to adult productivity are still poorly defined. These uncertainties underscore the need for longitudinal and mechanistic studies to clarify how early-life microbial assembly contributes to ruminant productivity.

## Growth

Early-life interventions could significantly improve growth performance in both dairy and meat-producing ruminants by shaping rumen development and digestive capacity. For example, introducing a starter diet to lambs as early as 7 days of age, combined with early weaning at 28 days, accelerates rumen maturation and improves growth performance, with this advantage persisting for up to two weeks after transition to a fattening diet [96]. Similarly, starter feeding from 10 days of age, particularly starch-rich formulations based on cassava, increases the abundance of acetate- and butyrate-producing bacterial taxa and starch-degrading ciliate protozoa in the rumen, thereby promoting rumen epithelial growth and functional development [36]. The type of solid feed also plays a critical role in directing rumen microbial establishment and tissue development. In lambs, starch-based corn–soybean starter diets induce the greatest rumen epithelial thickness, whereas fiber-rich alfalfa hay diets preferentially promote rumen muscular development, reflecting distinct microbial profiles associated with these diets [39]. Consistent with these findings, optimizing dietary protein in young lambs promotes higher average daily gain and improved feed conversion ratios while positively modulating the gastrointestinal microbiome toward fiber- and starch-degrading bacteria [97]. Together, these diet-driven microbial and morphological changes underpin efficient nutrient digestion and robust growth. Management strategies further modulate the trajectory of rumen development and growth outcomes. Comparative studies suggest that natural (dam) rearing can accelerate early rumen functional development, which may translate into higher growth rates during the subsequent fattening period, whereas overall body weight and carcass traits can converge when post-weaning diets and environments are standardized [98]. Importantly, evidence from artificially reared lambs demonstrates that the trajectory of rumen functional development is strongly driven by solid-feed intake and weaning management, and can be accelerated through appropriate step-down weaning strategies even in the absence of maternal rearing [99]. In addition to dietary and management interventions, microbial supplementation represents a complementary strategy for enhancing growth. Meta-analyses have shown that pre-weaning probiotic administration can increase body weight by approximately 1.99 kg and average daily gain by over 40 g/day in Holstein dairy calves, with these performance benefits associated with improvements in rumen fermentation parameters [51].

## Milk yield

Early-life nutritional and microbial interventions have also been associated with improved milk yield in adult dairy cows, primarily through effects on rumen development and nutrient metabolism. For example, supplementation of alfalfa hay to pre-weaning calves enhanced first-lactation milk production by improving rumen fermentation and microbial diversity, thereby optimizing nutrient extraction [100]. Similarly, early-life oral administration of fiber increased milk yield during the first month of lactation, an effect attributed to elevated VFA production, which stimulates rumen papillae development and accelerates functional maturation [101]. Mechanistically, these interventions enrich fibrolytic bacterial populations, enhance fiber breakdown, and maximize nutrient availability. However, not all early-life dietary or microbial interventions lead to persistent rumen microbiome changes or measurable long-term gains in milk yield. In a longitudinal study tracking calves from 2 weeks of age to first lactation, early-life diets (calf starter and/or corn silage) induced microbial differences at weaning, but these communities converged to an adult-like state after weaning, and the remaining microbiome variation was not associated with growth or first-lactation milk yield [102]. Similarly, microbial interventions, such as inoculating pre-weaning calves with rumen fluid from a feed-efficient adult cow, facilitated smoother energy utilization throughout the first lactation without influencing milk yield [16]. Discrepant findings may also reflect differences in experimental contrasts: intervention-versus-control studies often impose larger effect sizes than comparisons between multiple conventional feeding strategies, where shared post-weaning management can dilute early differences and obscure downstream lactational outcomes. Collectively, these findings suggest that early-life interventions have the potential to improve milk yield by accelerating rumen functional development and programming more efficient nutrient utilization during critical growth windows, thereby supporting sustained dairy productivity.

### Feed efficiency

Feed efficiency, a key component of sustainable ruminant production, often quantified as feed conversion ratio or residual feed intake, can be markedly improved by early-life nutritional and microbial interventions through their effects on rumen microbial assembly and function. For example, during the pre-weaning supplementation period, alfalfa hay supplementation promoted a cellulolytic rumen microbiota and reduced inflammation-associated taxa, which was accompanied by greater crude protein digestibility, ultimately improving overall nutrient utilization [100]. Similarly, dietary protein levels during early life strongly influence microbial community structure and metabolic outputs. In lambs, a medium-protein diet with a crude protein content of 112.0 g/kg dry matter improved the growth performance and nutrient efficiency of lambs through enriching specific fibrolytic and amylolytic taxa [97]. This microbial configuration supported higher daily weight gain and improved feed conversion ratios, while avoiding the inefficiencies and excess nitrogen emissions associated with overfeeding protein. Consistent with this framework, a recent synbiotic intervention in Holstein calves demonstrated that combined supplementation with xylooligosaccharides and *Bifidobacterium animalis* significantly increased average daily gain while reducing the feed-to-gain ratio, accompanied by enrichment of beneficial rumen taxa including *Bifidobacterium* and improved rumen fermentation profiles [103]. However, not all interventions yield consistent improvements in feed efficiency. A meta-analysis of probiotic supplementation in dairy calves reported no significant overall effect on feed efficiency and substantial heterogeneity among trials [104], indicating that microbial modulation must be context-dependent and nutritionally supported to translate into production benefits. These findings highlight how targeted nutritional modulation during early life can enrich functionally specialized taxa, optimize fermentation pathways, and reduce nitrogen waste, ultimately promoting both production efficiency and environmental sustainability.

### From early life microbial modulation to health outcomes

Early-life modulation of the microbiome plays a critical role in improving ruminant health by stabilizing the neonatal gut ecosystem, preventing diarrhea, and strengthening immune resilience, with these early health outcomes forming the biological foundation for long-term growth and performance throughout life **(**Fig. 1**)**. Neonatal calf diarrhea affects approximately 8.7% of beef calves and 25.5% of dairy calves within the first month of life, making it a leading cause of mortality and underscoring the need for timely interventions to prevent dysbiosis and its long-term consequences [105–107]. This condition is commonly associated with enteric pathogens such as *Escherichia coli*, *Salmonella*, *rotavirus*, *coronavirus*, and *Cryptosporidium* [108, 109].

### Diarrhea prevention

Since the seminal observation by Abe et al. (1995) linking higher *Bifidobacterium* abundance to reduced diarrhea incidence, this genus has been recognized as a key probiotic and an important pioneer colonizer in the neonatal GIT in ruminant health [110]. Calves with diarrhea typically exhibit lower microbial diversity and reduced *Bifidobacterium* levels compared with healthy peers, supporting its role as a microbial biomarker of gut health [65, 69–71]. Oral administration of *Bifidobacterium pseudolongum* can reduce diarrhea frequency and enhance gut barrier integrity, while multi-strain probiotic supplementation shortens symptom duration, particularly when administered at the onset of clinical signs [110, 111]. These protective effects are attributed to *Bifidobacterium*’s ability to metabolize milk oligosaccharides into acetate and lactate, which lower gut pH and inhibit pathogen growth, as well as its immunomodulatory functions such as promoting T regulatory cell differentiation [74, 77–80]. *Bifidobacterium* species encode diverse carbohydrate active enzymes that enable utilization of complex carbohydrates escaping upper gastrointestinal digestion, thereby supporting intestinal energy supply and epithelial integrity during early life [112]. Early colostrum mediated enrichment of *Bifidobacterium* is further associated with reduced opportunistic *Escherichia coli* and establishment of a protective neonatal gut microbial configuration [67]. Fecal microbiota transplantation (FMT) studies further suggest that *Bifidobacterium* may improve health by enhancing carbohydrate metabolism and VFA production [113]. However, persistence of *Bifidobacterium* post-weaning can be constrained by host genetics, diet, and maternal factors, highlighting the importance of optimizing both timing and delivery strategies of probiotic interventions.

Beyond bacterial taxa, viral components of the gut ecosystem also contribute to neonatal diarrhea dynamics. The bacteriophage *Escherichia* phage VpaE1_ev108 has been significantly associated with the progression of neonatal calf diarrhea [114], highlighting the tight coupling between viral populations and enteric pathogens. In parallel with probiotic approaches, bacteriophage supplementation has been explored as a precision strategy to selectively suppress pathogenic bacteria. Phage-mediated targeting of enteric pathogens has shown promise as an innovative therapeutic strategy for neonatal diarrhea management, with the potential to reduce reliance on conventional antibiotics, mitigate antimicrobial resistance, and alleviate disease while preserving gut microbiota homeostasis [115–117].

More holistic microbial interventions such as FMT have also shown therapeutic potential. In the FMT trial conducted in ruminants, administration of fecal microbes to pre-weaning calves improved diarrhea and restored gut microbial composition by increasing the relative abundance of Porphyromonadaceae, while metabolomic analysis revealed reduced fecal amino acid concentrations that strongly correlated with clinical remission [118]. Importantly, continuous follow-up over 24 months demonstrated that FMT not only improved gut health but also enhanced growth performance, suggesting durable benefits beyond the neonatal period [118]. Consistent results were observed in another FMT study in diarrheic calves, where approximately 70% of transplant recipients responded successfully; here, treatment efficacy was linked to donor–recipient microbial compatibility, particularly enrichment of *Selenomonas* and Veillonellaceae-associated metabolic networks, highlighting that both microbial and metabolic matching are key determinants of FMT success [119]. Importantly, not all FMT interventions yield beneficial outcomes. A large-scale calf trial reported that FMT did not prevent gastrointestinal disease and was associated with increased mortality, underscoring the risks of donor-derived pathogens and the need for rigorous donor screening, standardized preparation, and careful recipient selection [120]. These conflicting results highlight that the effectiveness of FMT depends heavily on the context and cannot yet be considered a generally reliable therapy.

Several other early-life microbial taxa have been implicated in modulating gut health and influencing diarrhea susceptibility in calves. A higher relative abundance of *Faecalibacterium*, a key anti-inflammatory commensal, has been associated with reduced diarrhea incidence in calves at one and three weeks of age [121]. Furthermore, specific early-life microbial signatures including *Trueperella*, *Streptococcus*, *Dorea*, uncultured Lachnospiraceae, *Ruminococcus* 2, and *Erysipelatoclostridium* have been identified as predictive biomarkers for diarrhea risk. A random forest machine learning model trained on these taxa achieved an 84.3% accuracy in distinguishing healthy from diarrheic calves, underscoring the potential for microbiome-based early detection and preventive management of gastrointestinal disease [54]. However, the ability to predict disease risk from microbial signatures does not necessarily translate into consistent therapeutic efficacy of microbiome-based interventions. Evidence from controlled trials and meta-analyses indicates that probiotic effects on calf health are highly heterogeneous. Reported benefits depend strongly on strain selection, dosage, formulation, and delivery method, and several studies have found no significant effects on diarrhea incidence, growth, or immune parameters. A meta-analysis confirmed substantial between-study variability and concluded that probiotic supplementation does not consistently confer measurable health benefits in calves across production systems [51].

### Immune resilience

Early microbial colonization is a key determinant of the host’s immune trajectory. Optimizing maternal microbiome and ensuring prompt colostrum feeding enhance passive immune transfer and promote early microbial diversification, thereby reducing susceptibility to enteric pathogens [71]. Accumulating evidence indicates that neonatal microbiota, shaped by breed, geographic origin, and maternal environment, has been linked to variations in disease susceptibility and immune responsiveness [69, 70, 122]. At the mechanistic level, targeted supplementation with probiotics and prebiotics during the neonatal period further strengthens epithelial barrier integrity by upregulating tight junction proteins and modulating inflammatory pathways [123], thereby minimizing infection risk. Consistently, Consistent with this framework, early-life transplantation of adult rumen microbiota attenuates inflammatory and immune-response pathways in the hindgut, underscoring the importance of caecal microbiota–host interactions in immune programming [124]. In contrast, excessive enteral starch supply during early development disrupts hindgut microbial–host homeostasis, compromises mucosal barrier integrity, and promotes T_H_2-skewed inflammatory responses through coordinated shifts in colonic microbiota, metabolites, and host immune signaling [125]. Collectively, these findings highlight that microbiota-targeted strategies during early life can promote immune stability and microbial resilience, reduce reliance on antimicrobial interventions, and improve calf health and welfare. By mitigating triggers for dysbiosis such as maternal separation, antibiotic exposure, or abrupt dietary changes, early-life microbial modulation establishes a durable foundation for lifelong health, resilience, and productivity in ruminants.

### From early life microbial modulation to methane mitigation

Enteric methane emissions from ruminants contribute about 18% of anthropogenic greenhouse gases, which poses a major barrier to sustainable livestock production [1]. Leveraging the plasticity of the early-life rumen microbiome offers a promising opportunity to transiently reduce methane emissions during early growth stages, even if long-term persistence of such effects remains uncertain. This section synthesizes the effects early-life microbial, dietary, and chemical interventions on methane emission, with attention to mechanisms, effectiveness, and persistence.

### Microbial interventions

Rumen methanogenesis is intrinsically linked to fermentation, where bacterial metabolism of feed produces VFAs and hydrogen, the latter serving as a key substrate for methanogenic archaea [126, 127]. One approach to inhibit methane production is to redirect hydrogen toward alternative metabolic pathways, such as propionate formation. By promoting these hydrogen-consuming pathways, microbial interventions can reprogram community assembly to reduce methanogenesis while maintaining or enhancing fermentation efficiency. Early-life supplementation with *Lacticaseibacillus rhamnosus* strains, for example, has been shown to induce sustained reductions in enteric methane emissions in growing dairy heifers [128]. Similarly, supplementation of *Saccharomyces cerevisiae* in legume-based diets in lambs has been reported to lower methane emissions [129]. Together, these biologically driven strategies function by modulating microbial succession during a window of developmental plasticity.

### Dietary interventions

Various dietary approaches have been explored as a means of shaping early life rumen microbiota in a way that reduces methane emissions. Lipid supplementation such as coconut oil reduces methane production by up to 82% in vitro, but depressed rumen fermentation and growth performance in vivo in goat kids, limiting its practical application [130]. Microalgae rich in docosahexaenoic acid and linseed oil produced minimal effects on methane emissions in goat kids and Holstein calves when supplemented both pre- and post-weaning [131, 132]. Jerusalem artichoke inulin at 10% did not reduce emissions when used alone [52], whereas a synbiotic combining Jerusalem artichoke with *Saccharomyces cerevisiae* significantly lowered methane emission by 15% in calves after 20 days of treatment with an increased number of total rumen prokaryotes [133]. Polyphenolic sugarcane extracts reduced methane by up to 24% in calves on pasture without compromising development, which suggests a promising, field-relevant approach [134].

### Anti-methanogenic compounds

Anti-methanogenic compounds reduce rumen methane production offering strategies for sustainable ruminant production. Coenzyme M analogs such as 3-nitrooxypropanol (3-NOP) inhibit methyl-coenzyme M reductase, and early-life administration in calves has been shown to induce a persistent reduction in methane emissions (approximately 10%) extending through the post-weaning period for at least one year, despite treatment cessation three weeks after weaning [135]. These effects were accompanied by shifts in fermentation patterns and microbial community structure. The CH_4_ reduction remained for seven month at 15% and then decreased [136]. No adverse effects on growth or physiological parameters were reported, suggesting that 3-NOP is safe for young calves at tested doses [135]. Halogenated compounds such as bromochloromethane (BCM) also demonstrated efficacy, with sequential studies showing reductions in methane emissions, alterations in archaeal colonization, and remodeling of rumen metabolomic profiles [137–139]. Seaweed-derived organobromines, such as from *Asparagopsis* spp., suppressed methanogenesis by up to 98.9% in vitro and in short-term animal trials [140], yet uncertainties remain regarding their long-term persistence, animal health implications, and food safety. Overall, while chemical inhibitors can induce substantial early-life methane suppression, further research is required to determine whether such effects persist across the full production cycle and to rigorously assess potential toxicological risks in young ruminants.

### Breeding strategies

Breeding represents a complementary approach to early-life interventions in reducing methane emissions. Methane output in cattle shows moderate heritability (estimates ranging from 0.12 to 0.45), enabling genetic progress [141, 142]. Both host genotype and rumen microbial composition contribute to methane yield, suggesting that selection should consider favorable microbial profiles alongside conventional production traits [143, 144]. Importantly, host genetics not only influence adult traits but also shape microbial colonization trajectories in early life [31, 145]. For instance, calves with high-efficiency genotypes tend to harbor microbial communities with lower methanogenic potential from a young age, leading to improved feed conversion and reduced emissions later in life [146]. Integrating early-life microbiota data into breeding programs may thus enhance selection accuracy for environmentally favorable phenotypes. Tools such as mid-infrared reflectance spectroscopy of milk are advancing phenotyping in dairy herds, enabling earlier and more precise selection for low-methane cattle [147]. With sustained efforts in genotyping, phenotyping, and microbiome monitoring from birth onward, methane emissions could be reduced by up to 45% across generations [148]. Together, these findings highlight the potential of breeding strategies, particularly those informed by early-life microbiome signatures, to achieve long-term and heritable reductions in enteric methane emissions.

### Concluding remarks and future perspectives

Early-life gut microbiome interventions hold considerable promise for advancing sustainable ruminant production; however, several critical challenges remain. Evidence from longitudinal and comparative studies indicates that these benefits are not universally persistent, with outcomes such as methane mitigation frequently diminishing during post-weaning maturation due to microbial community convergence. This ecological homogenization can override the initially induced microbial configurations, thereby compromising the long-term efficacy of interventions. Beyond microbial community convergence, ecological mechanisms such as functional redundancy and priority effects during early colonization can further dampen the persistence of early microbial perturbations, as overlapping metabolic functions and later community assembly processes promote the re-establishment of similar functional states. Furthermore, variability in intervention protocols, environmental exposures, and farm-level management practices limits reproducibility and impedes broad implementation. Moreover, potential trade-offs between environmental sustainability objectives and key production traits, including growth performance, milk yield, and reproductive efficiency, necessitate continuous and rigorous evaluation to ensure that ecological gains do not come at the expense of animal productivity or welfare.

**Fig. 2:**
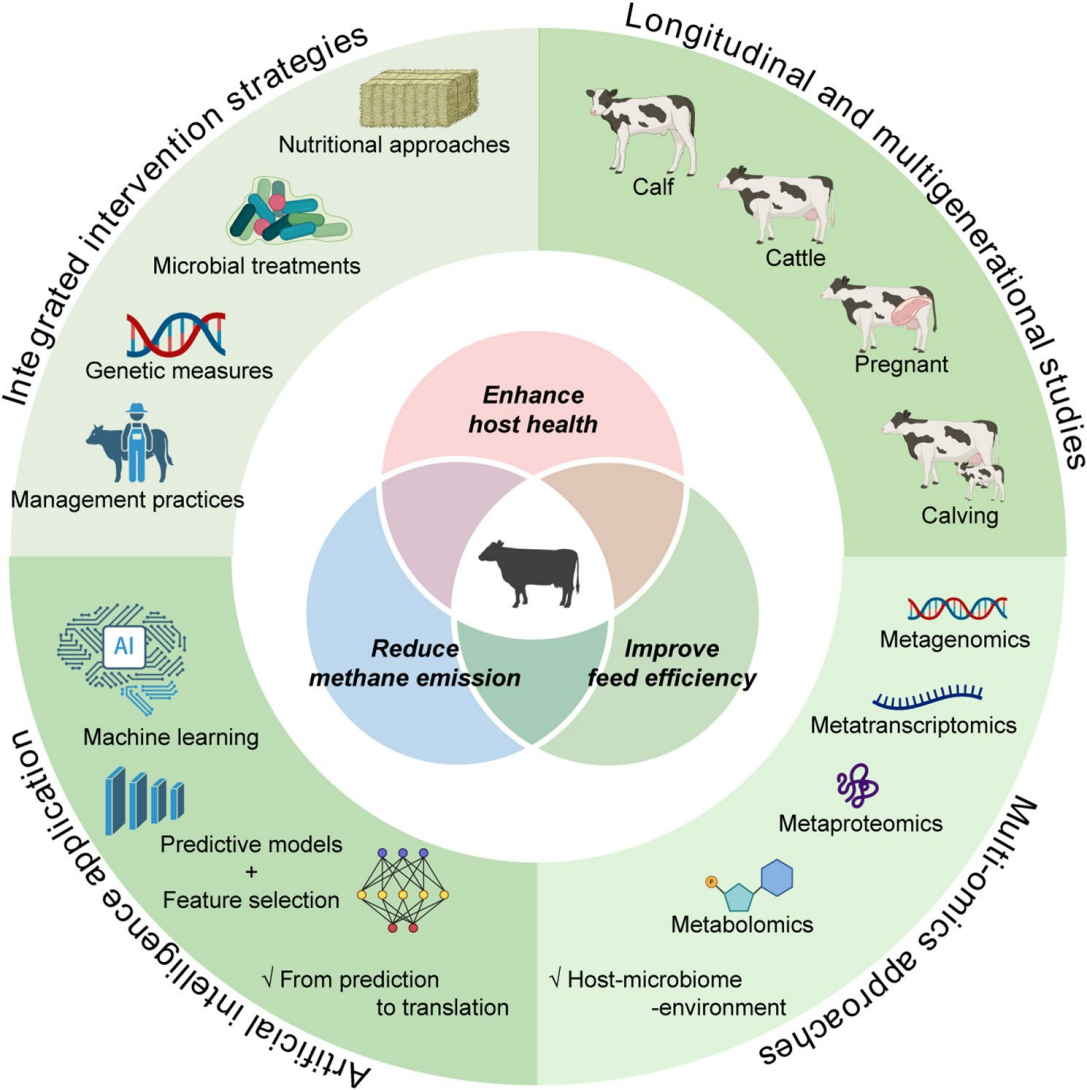
Schematic overview of four priority directions for advancing early-life gut microbiome interventions toward sustainable ruminant production: (1) integration of nutritional, microbial, genetic, and management strategies to enhance host health, feed efficiency, and methane reduction; (2) implementation of longitudinal and multigenerational studies to evaluate the persistence and potential heritability of intervention outcomes across developmental stages and production cycles; (3) application of multi-omics and systems biology to uncover host–microbiome–environment interactions and identify predictive microbial and metabolic signatures; and (4) incorporation of artificial intelligence, including machine learning and predictive modeling, to support precision microbiome management and facilitate scalable application in commercial systems. Created in BioRender

Current evidence supports the effectiveness of integrated intervention strategies that combine nutritional approaches (such as high-fiber or functional feeds), microbial treatments (including probiotics and methane inhibitors), genetic measures (such as genomic selection for low-methane phenotypes), and management practices (such as preserving maternal contact during early life) (Fig. 2). Despite this, robust longitudinal and multigenerational studies remain scarce, limiting our ability to assess the persistence and potential transgenerational inheritance of early-life intervention outcomes. Such studies are essential for identifying critical developmental windows, validating long-term phenotypic impacts, and designing interventions that can maintain effectiveness across multiple production cycles and environmental conditions.

A deeper mechanistic understanding will require the integration of metagenomics, metatranscriptomics, metaproteomics, and metabolomics within a systems biology framework to disentangle the host–microbiome–environment interactions that underpin intervention outcomes. Future research should leverage these multi-omics approaches to identify robust microbial or metabolic biomarkers predictive of early-life intervention success, with a shift from targeting individual species toward modulating core functional modules or regulatory networks conserved across microbial communities. These approaches can reveal the microbial taxa, metabolic pathways, and regulatory networks responsible for improvements in feed efficiency, reductions in methane emissions, and enhancements in animal health.

Looking ahead, artificial intelligence offers substantial and still largely untapped opportunities in microbiome research. Deep learning–based protein language models such as ESM2 [149] and structure prediction frameworks including AlphaFold3 [150] have demonstrated how artificial intelligence can enhance functional annotation of metagenomic sequences. Similarly, supervised learning tools have been applied to predict CAZymes, biosynthetic gene clusters, antimicrobial peptides, and antimicrobial resistance genes, illustrating the capacity of artificial intelligence to move beyond descriptive profiling toward functional interpretation [151–153]. Supervised models integrating early-life microbiome data, host genomic information, and longitudinal phenotypes could enable early prediction of methane emissions, feed efficiency, or health, creating opportunities for proactive, stage-specific intervention. When combined with sensor-derived production data and embedded into farm-level decision-support platforms, interpretable machine learning frameworks could support genotype-tailored nutritional adjustments (e.g., dietary protein optimization) or targeted microbial supplementation during early development. Thus, artificial intelligence represents a powerful yet still emerging toolkit for translating microbiome knowledge into practical precision livestock management.

Future advances will necessitate hybrid analytical frameworks that harness multi-omics datasets, predictive modelling, causal inference, and targeted culturing or synthetic community approaches to propel precision microbiome engineering in livestock. Complementary experimental platforms, such as rumen/gut simulation systems or rumen/gut-derived organoids, could provide controlled environments to screen and validate candidate interventions and microbial consortia prior to large-scale animal trials, thereby accelerating translation while reducing cost and variability. Collectively these approaches will facilitate the development of interventions that are both biologically effective and readily adaptable to diverse genetic backgrounds, production systems, and environmental conditions. Addressing these priorities will be essential for translating early-life microbiome modulation into practical, durable, and environmentally sustainable solutions for global ruminant production.

## Data Availability

No datasets were generated or analysed during the current study.

## Abbreviations

VFA: Volatile fatty acid
GIT: Gastrointestinal tract
16S rRNA: 16 S ribosomal ribonucleic acid
ASV: Amplicon sequence variants
OTUs: operational taxonomic units
ATP: Adenosine triphosphate
FMT: Fecal microbiota transplantation
3-NOP: 3-nitrooxypropanol
BCM: Bromochloromethane
